# Crystal structure and Hirshfeld surface analysis of two imidazo[1,2-*a*]pyridine derivatives: *N*-*tert*-butyl-2-(4-meth­oxy­phen­yl)-5-methyl­imidazo[1,2-*a*]pyridin-3-amine and *N*-*tert*-butyl-2-[4-(di­methyl­amino)­phen­yl]imidazo[1,2-*a*]pyridin-3-amine

**DOI:** 10.1107/S2056989018016651

**Published:** 2018-11-30

**Authors:** G. Dhanalakshmi, Mala Ramanjaneyulu, Sathiah Thennarasu, S. Aravindhan

**Affiliations:** a Department of Physics, Misrimal Navajee Munoth Jain Engineering College, Chennai 600 097, India; b Organic & Bio-organic Chemistry Laboratory, Academy of Scientific and Innovative Research (AcSIR), CSIR-Central Leather Research Institute Adyar, Chennai 600 020, India; c Department of Physics, Presidency College (Autonomous), Chennai 600 005, India

**Keywords:** crystal structure, imidazole, imidazo[1,2-*a*]pyridine derivatives, N—H⋯N hydrogen bonding, C—H⋯π inter­actions, offset π–π inter­actions, Hirshfeld surface analysis, fingerprint plots

## Abstract

In the title imidazo[1,2-*a*]pyridine derivatives, *N*-*tert*-butyl-2-(4-meth­oxy­phen­yl)-5-methyl­imidazo[1,2-*a*]pyridin-3-amine, C_19_H_23_N_3_O, (I), and *N*-*tert*-butyl-2-[4-(di­methyl­amino)­phen­yl]imidazo[1,2-*a*]pyridin-3-amine, C_19_H_24_N_4_, (II), the 4-meth­oxy­phenyl ring in (I) and the 4-(di­methyl­amino)­phenyl ring in (II) are inclined to the respective imidazole rings by 26.69 (9) and 31.35 (10)°. In the crystal of (I), mol­ecules are linked by N—H⋯N hydrogen bonds, forming chains propagating along the [001] direction. The chains are linked by C—H⋯π inter­actions, forming layers parallel to the (010) plane. In (II), the crystal packing also features N—H⋯N hydrogen bonds, which together with C—H⋯N hydrogen bonds link mol­ecules to form chains propagating along the *c*-axis direction. The chains are linked by C—H⋯π inter­actions to form layers parallel to the (100) plane. Inversion-related layers are linked by offset π–π inter­actions [inter­centroid distance = 3.577 (1) Å]. The inter­molecular inter­actions of both compounds were analyzed using Hirshfeld surface analysis and two-dimensional fingerprint plots.

## Abstract 2

In the title imidazo[1,2-*a*]pyridine derivatives, *N*-*tert*-butyl-2-(4-meth­oxy­phen­yl)-5-methyl­imidazo[1,2-*a*]pyridin-3-amine, (I), and *N*-*tert*-butyl-2-[4-(di­methyl­amino)­phen­yl]imidazo[1,2-*a*]pyridin-3-amine, (II), the 4-meth­oxy­phenyl ring in (I) and the 4-(di­methyl­amino)­phenyl ring in (II) are inclined to the mean planes of the respective imidazole rings by 26.69 (9) and 31.35 (10)°.

## Chemical context   

Imidazoles are heterocyclic compounds which show important pharmacological and biochemical properties. They exhibit anti-fungal (Banfi *et al.*, 2006 ▸), anti-bacterial (Jackson *et al.*, 2000 ▸), anti-tumour (Dooley *et al.*, 1992 ▸; Cui *et al.*, 2003 ▸), anti-protozoal (Biftu *et al.*, 2006 ▸), anti-herpes (Gudmundsson & Johns, 2007 ▸), anti-inflammatory (Rupert *et al.*, 2003 ▸), anti-ulcerative, anti-hypertensive, anti-histaminic and anti-helminthic properties (Spasov *et al.*, 1999 ▸). They also exhibit different therapeutic (Silvestre *et al.*, 1998 ▸; Lhassani *et al.*, 1999 ▸; Ertl *et al.*, 2000 ▸) and fluorescence properties (Kawai *et al.*, 2001 ▸; Abdullah, 2005 ▸). Imidazo[1,2-*a*]pyridines have been shown to be highly active against human cytomegalovirus and varicella-zoster virus (Gueffier *et al.*, 1998 ▸; Mavel *et al.*, 2002 ▸). In the present study, we report the synthesis, the single crystal X-ray diffraction studies, and Hirshfeld surface analysis of two new novel imidazole derivatives, *N*-*tert*-butyl-2-(4-meth­oxy­phen­yl)-5-methyl­imidazo[1,2-*a*]pyridin-3-amine, (I), and *N*-*tert*-butyl-2-[4-(di­methyl­amino)­phen­yl]imidazo[1,2-*a*]pyridin-3-amine, (II).
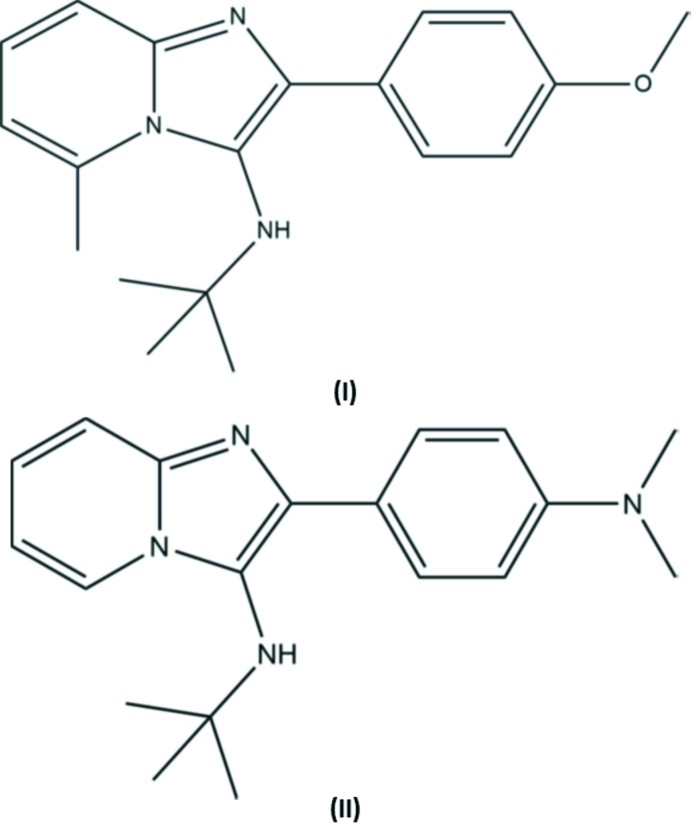


## Structural commentary   

**Figure 1:**
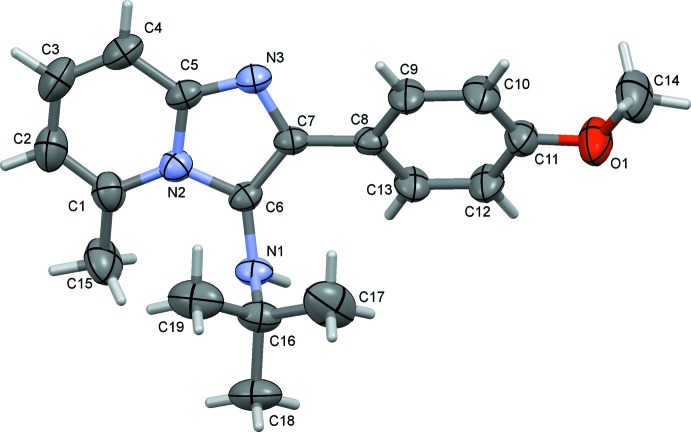
The mol­ecular structure of compound (I), with the atom labelling. Displacement ellipsoids are drawn at the 50% probability level.

**Figure 2:**
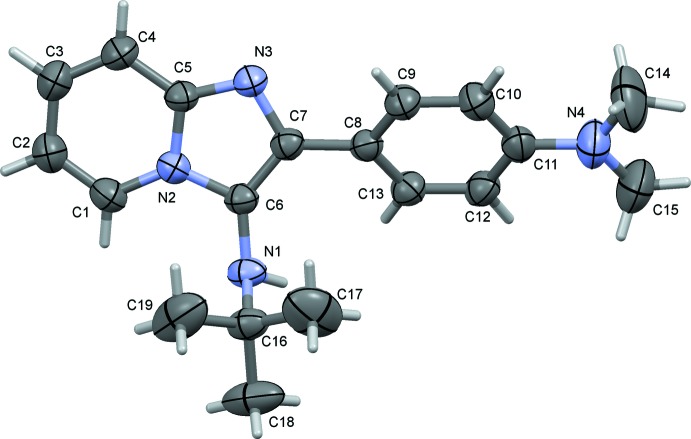
The mol­ecular structure of compound (II), with the atom labelling. Displacement ellipsoids are drawn at the 50% probability level.

**Figure 3:**
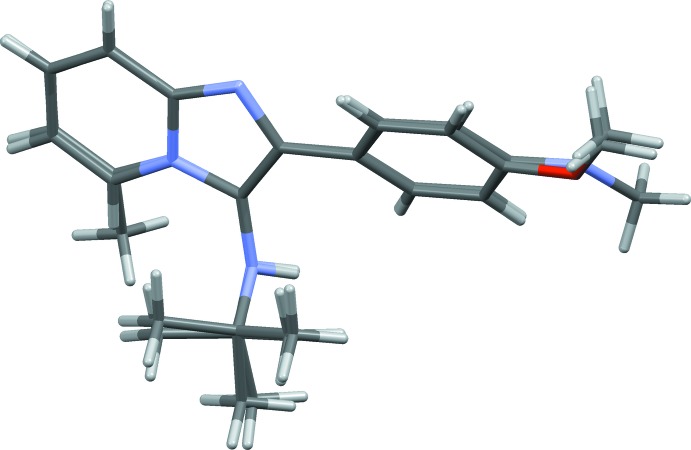
A structural overlap view of mol­ecules (I) and (II).

The mol­ecular structure of compound (I) is shown in Fig. 1 ▸, and that of compound (II) in Fig. 2 ▸. The overall conformation of the two mol­ecules is similar, as shown in the structural overlap drawing, Fig. 3 ▸. In compound (I), the imidazole ring system is planar with an r.m.s deviation of 0.062 Å and a maximum deviation of 0.071 (2) Å for atom C1. In compound (II), the imidazole ring system is planar with an r.m.s deviation of 0.029 Å and a maximum deviation of 0.031 (2) Å for atom N2. In (I) the pyridine ring (N2/C1–C5) of the imidazole ring system makes a dihedral angle of 4.91 (11)° with the five-membered ring (N2/N3/C5–C7), while the corresponding angle in (II) is 2.90 (13)°. In both compounds, the difference in endocyclic angles [129.27 (19)° for bond angle C4—C5—N3 and 132.33 (17)° for bond angle C1—N2—C6 in compound (I), and 131.1 (2) and 130.4 (2)°, respectively, in compound (II)] of the imidazole ring systems are due to the merging of five- and six-membered rings and the strain is taken up by angular distortion rather than by bond length distortion.

The dihedral angle between the pyridine (N2/C1–C5) and the benzene (C8–C13) rings is 25.04 (10)° in (I) and 31.11 (12) ° in (II). In (I) the meth­oxy group (C11/O1/C14) lies in the plane of the benzene ring (C8–C13) to which it is attached, with a dihedral angle of 0.6 (2)°. In (II) the di­methyl­amine group (N4/C14/C15) also lies close to the plane of the benzene ring (C8–C13) with a dihedral angle of 1.42 (19)°. The dihedral angle between atoms N1/C16/C18 and the imidazole ring mean plane is 80.28 (19)° in (I) and 84.6 (2)° in (II). The sum of the bond angles around atom N2 is 359.87 ° in (I), and the sums around atoms N2 and N4 in (II) are 359.85 and 360.0°, respectively, indicating *sp* ^2^ hybridization. In compound (I) the torsion angles C10—C9—C8—C7 and C18—C16—N1—C6 are −178.9 (2) and 170.52 (18)°, respectively, while the corresponding torsion angles in compound (II) are −177.9 (2) and 179.4 (2)°, respectively. This shows that for both compounds the imidazole ring is (−) anti­periplanar with the benzene ring and (+) anti­periplanar with the side-chain atoms N1, C16 and C18.

## Supra­molecular features   

**Figure 4:**
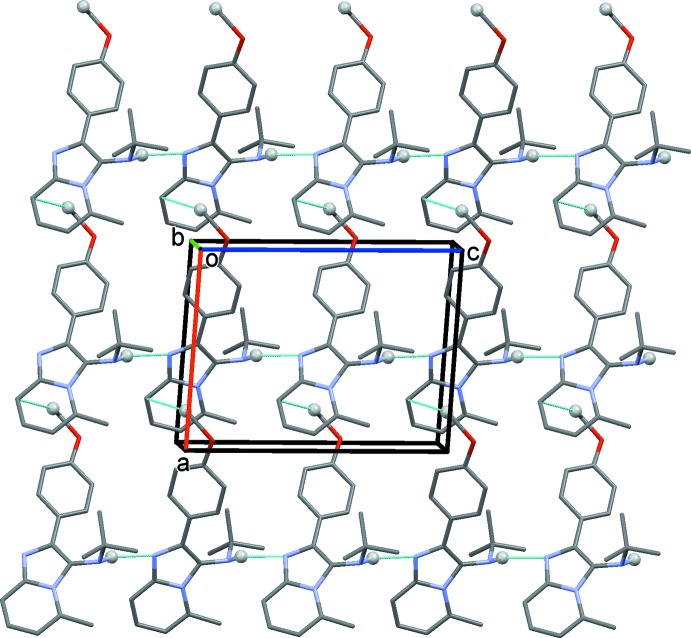
The crystal packing of compound (I) viewed along the *b* axis, showing the inter­molecular N—H⋯N hydrogen bonds as dashed lines (Table 1 ▸). The C—H⋯π inter­actions are also represented by cyan dashed lines (Table 1 ▸).

In the crystal of (I), mol­ecules are linked by N1—H1*A*⋯N3^i^ hydrogen bonds (Table 1 ▸), forming *C*(8) chains propagating along the *c*-axis direction, as shown in Fig. 4 ▸. The chains are linked by C—H⋯π inter­actions, forming layers lying parallel to the *ac* plane (Fig. 4 ▸, Table 1 ▸).

**Figure 5:**
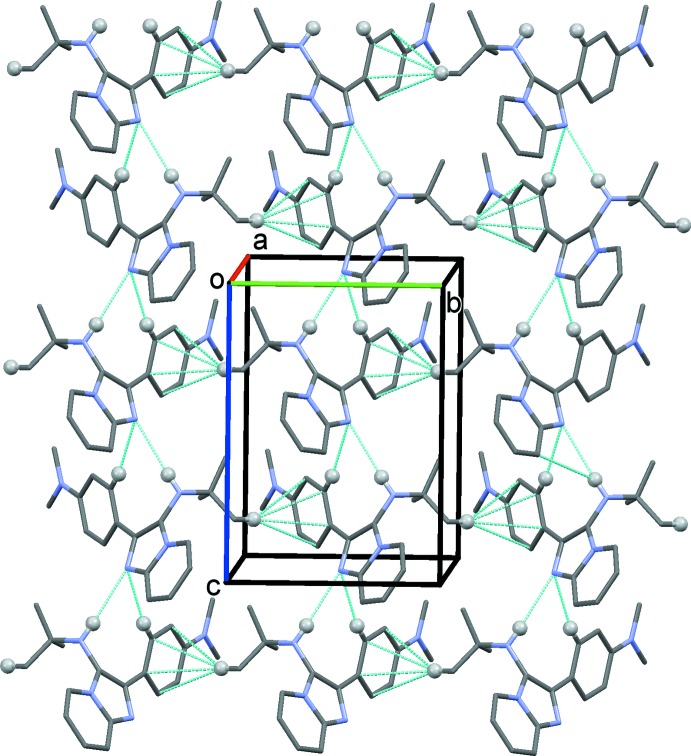
The crystal packing of compound (II) viewed along the *a* axis, showing the inter­molecular N—H⋯N and C—H⋯N hydrogen bonds and C—H⋯π inter­actions as dashed lines (Table 2 ▸).

In the crystal of (II), mol­ecules are linked by N1—H1*A*⋯N3^i^ and C13—H13⋯N3^i^ hydrogen bonds (Table 2 ▸), forming chains propagating along the [001] direction, as shown in Fig. 5 ▸. The chains are also linked by C—H⋯π inter­actions, forming layers lying parallel to the *bc* plane (Fig. 5 ▸, Table 2 ▸). Inversion-related layers are linked by offset π–π inter­actions involving the pyridine ring of the imidazole ring system: *Cg*2⋯*Cg*2^iii^ = 3.577 (1) Å, *Cg*2 is the centroid of the pyridine ring (N2/C1–C5), α = 0.0 (1)°, β = 22.3°, inter­planar distance = 3.309 (1) Å, offset = 1.357 Å; symmetry code (iii) −*x* + 1, −*y* + 1, −*z* + 1.

## Hirshfeld Surface Analysis   

**Figure 6:**
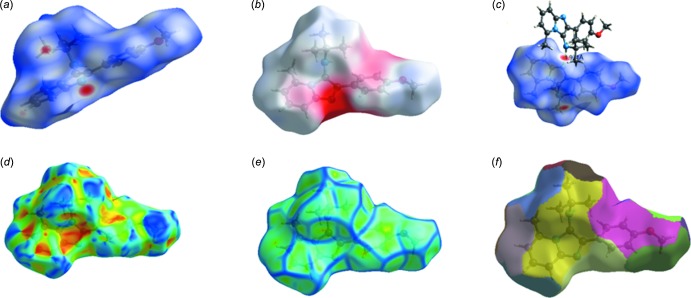
View of the Hirshfeld surface for compound (I), mapped over: (*a*) *d*_norm_; (*b*) electrostatic potential; (*c*) inter­molecular contacts; (*d*) shape index; (*e*) curvature; (*f*) fragment patches.

**Figure 7:**
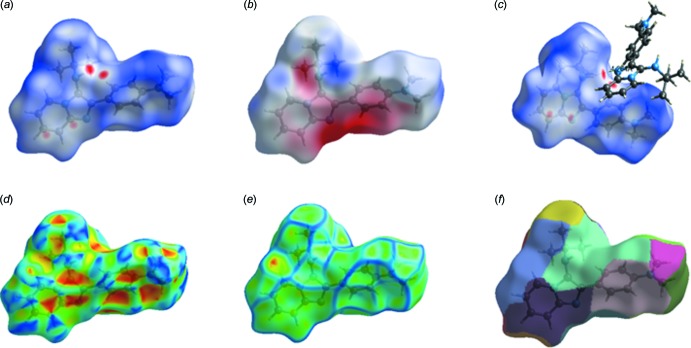
View of the Hirshfeld surface for compound (II), mapped over: (*a*) *d*_norm_; (*b*) electrostatic potential; (*c*) inter­molecular contacts; (*d*) shape index; (*e*) curvature; (*f*) fragment patches.

**Figure 8:**
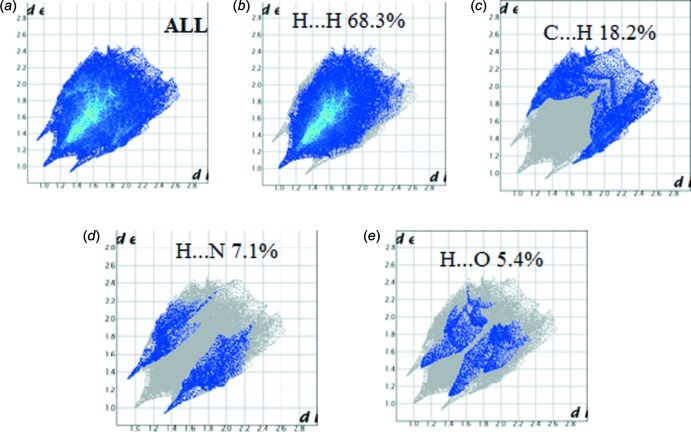
Two-dimensional fingerprint plots for compound (I): (*a*) all inter­molecular inter­actions; (*b*) H⋯H contacts; (*c*) C⋯·H contacts; (*d*) H⋯ N contacts; (*e*) H⋯O contacts.

**Figure 9:**
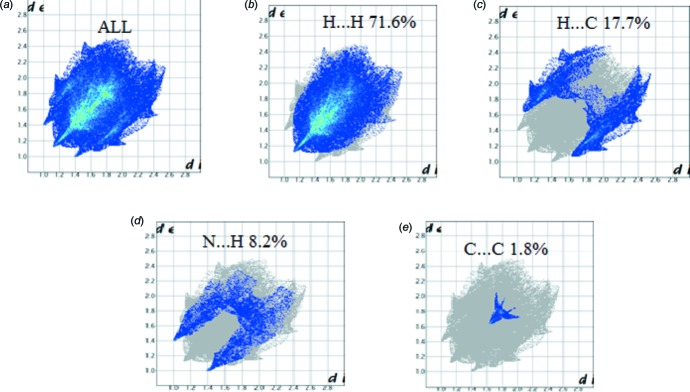
Two-dimensional fingerprint plots for compound (II): (*a*) all inter­molecular inter­actions; (*b*) H⋯H contacts; (*c*) H⋯C contacts; (*d*) N⋯ H contacts; (*e*) C⋯C contacts.

Hirshfeld surface analysis was used to qu­antify the inter­molecular contacts of the title compounds, using the software *CrystalExplorer17.5* (Turner *et al.*, 2017 ▸). The bright-red spots on the Hirshfeld surface mapped over *d*_norm_ [Fig. 6 ▸(*a*) and 7(*a*)], show the presence of N—H⋯N and C—H⋯ N inter­actions with neighbouring mol­ecules. The surfaces mapped over the electrostatic potential are illustrated in Fig. 6 ▸(*b*) and 7(*b*), while Fig. 6 ▸(*c*) and 7(*c*) show the inter­molecular contacts. The presence of red and blue triangles on the shape index map [Fig. 7 ▸(*d*)], indicates the presence of π–π stacking inter­actions in compound (II), and their absence in Fig. 6 ▸(*d*) shows that such inter­actions are absent in compound (I). The large flat region in Fig. 7 ▸(*e*), shown on the curvature map, confirms the presence of C—H⋯π inter­actions in compound (II). The fragment patches on the Hirshfeld surface [Figs. 6 ▸(*f*) and 7(*f*) ▸] show the coordination environments of the mol­ecules. The complete two-dimensional fingerprint plots are shown in Fig. 8 ▸(*a*) and 9(*a*). The H⋯H, C⋯H, N⋯H, C⋯N, H⋯O and C⋯C inter­actions are illustrated in Fig. 8 ▸(*b*)–8(*e*) for (I) and Fig. 9 ▸(*b*)–9(*e*) for (II). The H⋯H inter­actions make the largest contributions [Fig. 8 ▸(*b*) and 9(*b*)] to the overall Hirshfeld surfaces [68.3% for compound (I) and 71.6% for compound (II)]. The C⋯H inter­actions appear as two wings in the fingerprint plot [Fig. 8 ▸(*c*) and 9(*c*)], showing a contribution of 18.2% for compound (I) and 17.7% for compound (II) of the Hirshfeld surfaces. The contribution from the N⋯H contacts, corresponding to C—H⋯N inter­actions, is represented by a pair of sharp spikes with a contribution of 7.1% for compound (I) and 8.2% for compound (II) of the Hirshfeld surfaces [Fig. 8 ▸(*d*) and 9(*d*)]. The H⋯O contacts have a contribution of 5.4% of the Hirshfeld surface for compound (I). The C⋯C contacts, which refers to π–π inter­actions, contribute 1.8% of the Hirshfeld surfaces for compound (II). This can be seen in the shape of a butterfly at *d*_e_ = *d*_i_ 1.7Å [Fig. 9 ▸(*e*)].

## Database survey   

A search of the Cambridge Structural Database (CSD, version 5.39, last update August 2018; Groom *et al.*, 2016 ▸) revealed 29 hits for substructure imidazo[1,2-*a*]pyridin-3-amine and 16 hits for 5-methyl imidazo[1,2-*a*]pyridin-3-amine. Two compounds, (5-methyl­imidazo-[1,2-*a*]pyridin-2-yl)methanol (CSD refcode PONVUL; Elaatiaoui *et al.*, 2014 ▸), and ethyl 5-methyl­imidazo[1,2-*a*]pyridine-2-carboxyl­ate (DUSWOE; Yao *et al.*, 2010 ▸) are close analogues of compound (I). A third compound, (*E*)-2-phenyl-*N*-(thio­phen-2-yl­methyl­idene)-imidazo[1,2-*a*]pyridin-3-amine (OLEBOY; Elaatiaoui *et al.*, 2016 ▸), is a close analogue of compound (II). The crystal packing of compounds (I) and (II) are stabilized by N—H⋯N, C—H⋯N and C—H⋯π inter­actions, but the above mentioned crystal structures exhibit in general C—H⋯O, O—H⋯N and π–π inter­actions.

An inter­esting pyrazine analogue of compound (II) has been reported, *i.e. N*-*tert*-butyl-2-[4-(di­methyl­amino)­phen­yl]imidazo[1,2-*a*]pyrazin-3-amine (WIGKOO; Fatima *et al.*, 2013 ▸). Here the pyrazine and benzene rings are inclined to each other by 16.96 (7)°, compared to the corresponding dihedral angle of 31.11 (12)° involving the pyridine and benzene rings in (II). In the crystal, mol­ecules are linked *via* N—H⋯N hydrogen bonds, forming chains along [010], which in turn are linked by C—H⋯N hydrogen bonds forming layers parallel to the *ab* plane. This is very similar to the crystal-packing arrangement observed for compound (II).

## Synthesis and crystallization   

**Compound (I)**

5-Methyl-2-amino­pyridine (10 mmol) and 4-meth­oxy­benzaldehyde (1 eq.) were solubilized in ethanol. To this solution, *tert*-butyl isocyanide (1 eq.) and iodine (0.5 mmol %) were added. The reaction mixture was stirred at room temperature overnight. The white precipitate that had formed was filtered off and purified further using silica-gel column chromatography to give a white solid in 60% yield.

**Compound (II)**

2-Amino­pyridine (10 mmol) and 4-(di­methyl­amino) benzaldehyde (1 eq.) were solubilized in ethanol. To this solution, *tert*-butyl isocyanide (1 eq.) and iodine (0.5 mmol %) were added. The reaction mixture was stirred at room temperature overnight. The white precipitate that formed was filtered off and purified further using silica-gel column chromatography to give a yellow solid (yield 0.282 g, 91%).

***Spectroscopic data*** **:** NMR spectra were recorded on a Bruker 400 MHz NMR spectrophotometer in CdCl_3_ and chemical shifts were recorded in parts per million relative to tetra­methyl­silane (TMS), used as an inter­nal standard.

**Compound (I)**

^1^H NMR (400 MHz, CDCl_3_) δ = 8.57 (*ddd*, *J* = 4.9, 1.8, 0.9, 1H), 8.14 (*dt*, *J* = 8.0, 1.0, 1H), 7.77 (*td*, *J* = 7.7, 1.8, 1H), 7.40 (*d*, *J* = 9.0, 1H), 7.16 (*ddd*, *J* = 7.5, 4.9, 1.2, 1H), 7.01 (*dd*, *J* = 9.0, 6.7, 1H), 6.46–6.41 (*m*, 1H), 4.99 (*s*, 1H), 2.96 (*s*, 3H), 0.93 (*s*, 9H). ^13^C NMR (101 MHz, CDCl_3_) δ 155.31, 148.37, 142.86, 138.16, 137.65, 137.35, 136.54, 130.34, 124.28, 121.80, 121.79, 115.58, 113.91, 105.48, 57.20, 28.97, 20.21.

**Compound (II)**

^1^H NMR (CDCl_3_, 500 MHz): d_H_ 1.05 [*s*, 9H, –C(CH_3_)_3_], 2.97 [*s*, 6H, Ar-N(CH_3_)_2_], 6.69 (*t*, 1H, -Ar-H), 6.77 (*d*, 2H, *J* = 8.40 Hz, –Ar-H), 7.17 (*t*, 1H, –Ar-H, –Ar-H), 7.53 (*d*, 1H, *J* = 8.40 Hz, –Ar-H), 7.8 (*d*, 2H, *J* = 4.5 Hz, –Ar-H), 8.19 (*d*, 1H, *J* = 8.40 Hz, –Ar-H). ESI–MS: calculated for C_19_H_24_N_4_ [*M* + H]^+^ 308.2007; found: 308.27.

Crystals of compounds (I) and (II), suitable for X-ray diffraction analysis, were obtained by slow evaporation from ethyl alcohol (EtOH) solution at room temperature.

## Refinement   

Crystal data, data collection and structure refinement details are summarized in Table 3 ▸. For both compounds the NH H atoms were located in difference-Fourier maps and freely refined. The C-bound H atoms were included in calculated positions and treated as riding: C—H = 0.93–0.96 Å with *U*_iso_(H) = 1.5*U*_eq_(C-meth­yl) and 1.2*U*_eq_(C) for other H atoms.

## Supplementary Material

Crystal structure: contains datablock(s) I, II, Global. DOI: 10.1107/S2056989018016651/su5459sup1.cif

Structure factors: contains datablock(s) I. DOI: 10.1107/S2056989018016651/su5459Isup2.hkl

Structure factors: contains datablock(s) II. DOI: 10.1107/S2056989018016651/su5459IIsup3.hkl

CCDC references: 1838744, 1858376

Additional supporting information:  crystallographic information; 3D view; checkCIF report

## Figures and Tables

**Table 1:** Hydrogen-bond geometry (Å, °) for (I) *Cg*4 is the centroid of the imidazole ring system N2/N3/C1–C7.

*D*—H⋯*A*	*D*—H	H⋯*A*	*D*⋯*A*	*D*—H⋯*A*
N1—H1*A*⋯N3^i^	0.84 (2)	2.41 (2)	3.226 (2)	163.6 (19)
C14—H14*A*⋯*Cg*4^ii^	0.96	2.93	3.862 (3)	165

Symmetry codes: (i) 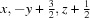; (ii) 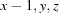.

**Table 2:** Hydrogen-bond geometry (Å, °) for (II) *Cg*3 is the centroid of benzene ring C8–C13.

*D*—H⋯*A*	*D*—H	H⋯*A*	*D*⋯*A*	*D*—H⋯*A*
N1—H1*A*⋯N3^i^	0.86 (3)	2.56 (3)	3.412 (3)	167 (2)
C13—H13⋯N3^i^	0.93	2.57	3.467 (3)	161
C19—H19*B*⋯*Cg*3^ii^	0.96	2.87	3.829 (4)	174

Symmetry codes: (i) 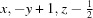; (ii) 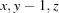.

**Table 3:** Experimental details

	(I)	(II)
Crystal data		
Chemical formula	C_19_H_23_N_3_O	C_19_H_24_N_4_
*M*_r_	309.40	308.42
Crystal system, space group	Monoclinic, *P*2_1_/*c*	Monoclinic, *C*2/*c*
Temperature (K)	296	296
*a*, *b*, *c* (Å)	9.2357 (7), 15.6388 (12), 11.984 (1)	34.9185 (14), 8.4656 (5), 11.8361 (6)
β (°)	93.998 (3)	91.061 (5)
*V* (Å^3^)	1726.7 (2)	3498.2 (3)
*Z*	4	8
Radiation type	Mo *K*α	Mo *K*α
μ (mm^−1^)	0.08	0.07
Crystal size (mm)	0.15 × 0.15 × 0.10	0.15 × 0.10 × 0.10

Data collection		
Diffractometer	Bruker Kappa APEXII CCD	Bruker Kappa APEXII CCD
Absorption correction	Multi-scan (*SADABS*; Bruker, 2016 ▸)	Multi-scan (*SADABS*; Bruker, 2016 ▸)
*T*_min_, *T*_max_	0.552, 0.746	0.697, 0.745
No. of measured, independent and observed [*I* > 2σ(*I*)] reflections	16458, 3208, 2109	32313, 3259, 1834
*R*_int_	0.044	0.071
(sin θ/λ)_max_ (Å^−1^)	0.606	0.606

Refinement		
*R*[*F* ^2^ > 2σ(*F* ^2^)], *wR*(*F* ^2^), *S*	0.047, 0.119, 1.03	0.049, 0.159, 1.02
No. of reflections	3208	3259
No. of parameters	218	218
H-atom treatment	H atoms treated by a mixture of independent and constrained refinement	H atoms treated by a mixture of independent and constrained refinement
Δρ_max_, Δρ_min_ (e Å^−3^)	0.15, −0.13	0.22, −0.18

Computer programs: *APEX2*, *SAINT* and *XPREP* (Bruker, 2016 ▸), *SHELXT2014* (Sheldrick, 2015*a* ▸), *SHELXL2018* (Sheldrick, 2015*b* ▸), *ORTEP-3 for Windows* (Farrugia, 2012 ▸), *Mercury* (Macrae *et al.*, 2008 ▸), *PLATON* (Spek, 2009 ▸) and *publCIF* (Westrip, 2010 ▸).

## supplementary crystallographic information

### N-tert-Butyl-2-(4-methoxyphenyl)-5-methylimidazo[1,2-a]pyridin-3-amine (I) Crystal data

**Table:**

C_19_H_23_N_3_O	*F*(000) = 664
*M**_r_* = 309.40	*D*_x_ = 1.190 Mg m^−^^3^
Monoclinic, *P*2_1_/*c*	Mo *K*α radiation, λ = 0.71073 Å
*a* = 9.2357 (7) Å	Cell parameters from 3732 reflections
*b* = 15.6388 (12) Å	θ = 2.6–29.2°
*c* = 11.984 (1) Å	µ = 0.08 mm^−^^1^
β = 93.998 (3)°	*T* = 296 K
*V* = 1726.7 (2) Å^3^	Block, colourless
*Z* = 4	0.15 × 0.15 × 0.10 mm

### N-tert-Butyl-2-(4-methoxyphenyl)-5-methylimidazo[1,2-a]pyridin-3-amine (I) Data collection

**Table:**

Bruker Kappa APEXII CCD diffractometer	3208 independent reflections
Radiation source: fine-focus sealed tube	2109 reflections with *I* > 2σ(*I*)
Graphite monochromator	*R*_int_ = 0.044
ω and φ scan	θ_max_ = 25.5°, θ_min_ = 2.2°
Absorption correction: multi-scan (SADABS; Bruker, 2016)	*h* = −11→11
*T*_min_ = 0.552, *T*_max_ = 0.746	*k* = −18→18
16458 measured reflections	*l* = −14→13

### N-tert-Butyl-2-(4-methoxyphenyl)-5-methylimidazo[1,2-a]pyridin-3-amine (I) Refinement

**Table:**

Refinement on *F*^2^	Secondary atom site location: difference Fourier map
Least-squares matrix: full	Hydrogen site location: mixed
*R*[*F*^2^ > 2σ(*F*^2^)] = 0.047	H atoms treated by a mixture of independent and constrained refinement
*wR*(*F*^2^) = 0.119	*w* = 1/[σ^2^(*F*_o_^2^) + (0.0386*P*)^2^ + 0.6929*P*] where *P* = (*F*_o_^2^ + 2*F*_c_^2^)/3
*S* = 1.03	(Δ/σ)_max_ < 0.001
3208 reflections	Δρ_max_ = 0.15 e Å^−^^3^
218 parameters	Δρ_min_ = −0.13 e Å^−^^3^
0 restraints	Extinction correction: SHELXL2018 (Sheldrick, 2015b), Fc^*^=kFc[1+0.001xFc^2^λ^3^/sin(2θ)]^-1/4^
Primary atom site location: structure-invariant direct methods	Extinction coefficient: 0.0063 (11)

### N-tert-Butyl-2-(4-methoxyphenyl)-5-methylimidazo[1,2-a]pyridin-3-amine (I) Special details

**Table:**

Geometry. All esds (except the esd in the dihedral angle between two l.s. planes) are estimated using the full covariance matrix. The cell esds are taken into account individually in the estimation of esds in distances, angles and torsion angles; correlations between esds in cell parameters are only used when they are defined by crystal symmetry. An approximate (isotropic) treatment of cell esds is used for estimating esds involving l.s. planes.

### N-tert-Butyl-2-(4-methoxyphenyl)-5-methylimidazo[1,2-a]pyridin-3-amine (I) Fractional atomic coordinates and isotropic or equivalent isotropic displacement parameters (Å^2^)

**Table:**

	*x*	*y*	*z*	*U*_iso_*/*U*_eq_	
O1	−0.02423 (18)	0.88980 (13)	0.62308 (14)	0.0800 (6)	
N1	0.58139 (18)	0.64812 (11)	0.72686 (14)	0.0385 (4)	
H1A	0.560 (2)	0.6854 (13)	0.7736 (17)	0.045 (6)*	
N2	0.71475 (16)	0.66024 (10)	0.55928 (13)	0.0381 (4)	
N3	0.55442 (18)	0.72815 (11)	0.43982 (13)	0.0417 (4)	
C1	0.8497 (2)	0.62484 (14)	0.59167 (19)	0.0480 (6)	
C2	0.9389 (2)	0.60796 (16)	0.5092 (2)	0.0618 (7)	
H2	1.028199	0.582474	0.528113	0.074*	
C3	0.9016 (3)	0.62747 (17)	0.3962 (2)	0.0633 (7)	
H3	0.963493	0.611842	0.341717	0.076*	
C4	0.7768 (2)	0.66868 (14)	0.36672 (18)	0.0524 (6)	
H4	0.754319	0.684394	0.292687	0.063*	
C5	0.6809 (2)	0.68762 (13)	0.44948 (16)	0.0401 (5)	
C6	0.5944 (2)	0.68161 (12)	0.61971 (15)	0.0345 (5)	
C7	0.5016 (2)	0.72552 (12)	0.54407 (15)	0.0361 (5)	
C8	0.3630 (2)	0.76695 (12)	0.56396 (15)	0.0367 (5)	
C9	0.2546 (2)	0.77465 (15)	0.47894 (17)	0.0516 (6)	
H9	0.270036	0.752223	0.408881	0.062*	
C10	0.1244 (2)	0.81454 (16)	0.49472 (18)	0.0574 (6)	
H10	0.053459	0.818460	0.435930	0.069*	
C11	0.0997 (2)	0.84844 (15)	0.59730 (18)	0.0512 (6)	
C12	0.2067 (2)	0.84190 (15)	0.68339 (18)	0.0540 (6)	
H12	0.190965	0.864567	0.753249	0.065*	
C13	0.3361 (2)	0.80227 (14)	0.66681 (17)	0.0463 (5)	
H13	0.407187	0.799021	0.725600	0.056*	
C14	−0.1377 (3)	0.8996 (2)	0.5385 (2)	0.0848 (9)	
H14A	−0.170048	0.844251	0.512568	0.127*	
H14B	−0.102994	0.931447	0.477349	0.127*	
H14C	−0.217011	0.929636	0.568295	0.127*	
C15	0.8957 (3)	0.61354 (19)	0.7129 (2)	0.0712 (8)	
H15A	0.992141	0.590353	0.720247	0.107*	
H15B	0.830116	0.575101	0.746182	0.107*	
H15C	0.894405	0.667913	0.749953	0.107*	
C16	0.4969 (2)	0.56772 (13)	0.73775 (17)	0.0478 (5)	
C17	0.3348 (3)	0.58040 (18)	0.7103 (3)	0.0830 (9)	
H17A	0.318020	0.596991	0.633344	0.124*	
H17B	0.299696	0.624313	0.757429	0.124*	
H17C	0.284589	0.527895	0.722769	0.124*	
C18	0.5237 (3)	0.53949 (16)	0.85894 (19)	0.0676 (7)	
H18A	0.491235	0.583342	0.907398	0.101*	
H18B	0.625574	0.529685	0.875337	0.101*	
H18C	0.471174	0.487645	0.870558	0.101*	
C19	0.5532 (3)	0.50019 (15)	0.6600 (2)	0.0712 (8)	
H19A	0.654518	0.490342	0.679215	0.107*	
H19B	0.540247	0.519762	0.584077	0.107*	
H19C	0.500409	0.447913	0.667862	0.107*	

### N-tert-Butyl-2-(4-methoxyphenyl)-5-methylimidazo[1,2-a]pyridin-3-amine (I) Atomic displacement parameters (Å^2^)

**Table:**

	*U*^11^	*U*^22^	*U*^33^	*U*^12^	*U*^13^	*U*^23^
O1	0.0547 (10)	0.1337 (17)	0.0507 (11)	0.0372 (11)	−0.0036 (8)	−0.0104 (11)
N1	0.0478 (10)	0.0425 (10)	0.0246 (9)	−0.0031 (8)	−0.0024 (7)	−0.0012 (8)
N2	0.0392 (9)	0.0403 (9)	0.0346 (10)	−0.0010 (7)	0.0019 (7)	−0.0022 (7)
N3	0.0473 (10)	0.0488 (10)	0.0290 (9)	0.0023 (8)	0.0035 (7)	0.0027 (8)
C1	0.0412 (12)	0.0509 (13)	0.0513 (14)	0.0026 (10)	−0.0018 (10)	−0.0050 (11)
C2	0.0425 (13)	0.0714 (17)	0.0720 (18)	0.0081 (12)	0.0084 (12)	−0.0068 (14)
C3	0.0550 (15)	0.0765 (17)	0.0612 (17)	0.0001 (13)	0.0240 (13)	−0.0099 (14)
C4	0.0582 (15)	0.0613 (15)	0.0392 (13)	−0.0063 (12)	0.0147 (11)	−0.0027 (11)
C5	0.0472 (12)	0.0430 (11)	0.0307 (11)	−0.0073 (10)	0.0058 (9)	−0.0019 (9)
C6	0.0374 (10)	0.0398 (11)	0.0263 (10)	−0.0045 (9)	0.0019 (8)	−0.0040 (8)
C7	0.0416 (11)	0.0407 (11)	0.0257 (10)	−0.0025 (9)	−0.0001 (8)	0.0006 (9)
C8	0.0400 (11)	0.0428 (11)	0.0267 (10)	−0.0002 (9)	−0.0013 (8)	0.0032 (9)
C9	0.0536 (13)	0.0747 (16)	0.0256 (11)	0.0113 (12)	−0.0037 (10)	−0.0055 (11)
C10	0.0494 (13)	0.0846 (17)	0.0363 (13)	0.0130 (12)	−0.0114 (10)	−0.0019 (12)
C11	0.0428 (12)	0.0695 (15)	0.0410 (13)	0.0112 (11)	0.0000 (10)	0.0011 (11)
C12	0.0516 (13)	0.0801 (16)	0.0300 (12)	0.0137 (12)	0.0002 (10)	−0.0098 (11)
C13	0.0457 (12)	0.0609 (14)	0.0307 (12)	0.0066 (10)	−0.0090 (9)	−0.0043 (10)
C14	0.0514 (15)	0.127 (3)	0.074 (2)	0.0277 (16)	−0.0128 (14)	−0.0110 (18)
C15	0.0511 (14)	0.097 (2)	0.0626 (17)	0.0198 (14)	−0.0146 (12)	−0.0050 (15)
C16	0.0568 (13)	0.0468 (12)	0.0386 (12)	−0.0101 (11)	−0.0062 (10)	0.0059 (10)
C17	0.0623 (17)	0.0747 (18)	0.109 (2)	−0.0249 (14)	−0.0135 (16)	0.0225 (17)
C18	0.097 (2)	0.0635 (16)	0.0422 (14)	−0.0124 (14)	0.0028 (13)	0.0133 (12)
C19	0.112 (2)	0.0494 (14)	0.0506 (15)	−0.0149 (15)	−0.0056 (14)	−0.0033 (12)

### N-tert-Butyl-2-(4-methoxyphenyl)-5-methylimidazo[1,2-a]pyridin-3-amine (I) Geometric parameters (Å, º)

**Table:**

O1—C11	1.368 (3)	C10—C11	1.372 (3)
O1—C14	1.414 (3)	C10—H10	0.9300
N1—C6	1.400 (2)	C11—C12	1.382 (3)
N1—C16	1.490 (3)	C12—C13	1.373 (3)
N1—H1A	0.84 (2)	C12—H12	0.9300
N2—C1	1.394 (3)	C13—H13	0.9300
N2—C5	1.398 (2)	C14—H14A	0.9600
N2—C6	1.409 (2)	C14—H14B	0.9600
N3—C5	1.327 (2)	C14—H14C	0.9600
N3—C7	1.373 (2)	C15—H15A	0.9600
C1—C2	1.355 (3)	C15—H15B	0.9600
C1—C15	1.495 (3)	C15—H15C	0.9600
C2—C3	1.407 (3)	C16—C18	1.522 (3)
C2—H2	0.9300	C16—C19	1.523 (3)
C3—C4	1.346 (3)	C16—C17	1.523 (3)
C3—H3	0.9300	C17—H17A	0.9600
C4—C5	1.407 (3)	C17—H17B	0.9600
C4—H4	0.9300	C17—H17C	0.9600
C6—C7	1.385 (3)	C18—H18A	0.9600
C7—C8	1.469 (3)	C18—H18B	0.9600
C8—C9	1.383 (3)	C18—H18C	0.9600
C8—C13	1.389 (3)	C19—H19A	0.9600
C9—C10	1.380 (3)	C19—H19B	0.9600
C9—H9	0.9300	C19—H19C	0.9600
			
C11—O1—C14	118.61 (19)	C13—C12—H12	119.7
C6—N1—C16	118.36 (16)	C11—C12—H12	119.7
C6—N1—H1A	112.9 (14)	C12—C13—C8	121.27 (19)
C16—N1—H1A	112.4 (14)	C12—C13—H13	119.4
C1—N2—C5	121.38 (17)	C8—C13—H13	119.4
C1—N2—C6	132.33 (17)	O1—C14—H14A	109.5
C5—N2—C6	106.16 (15)	O1—C14—H14B	109.5
C5—N3—C7	105.83 (16)	H14A—C14—H14B	109.5
C2—C1—N2	116.8 (2)	O1—C14—H14C	109.5
C2—C1—C15	122.6 (2)	H14A—C14—H14C	109.5
N2—C1—C15	120.36 (19)	H14B—C14—H14C	109.5
C1—C2—C3	122.6 (2)	C1—C15—H15A	109.5
C1—C2—H2	118.7	C1—C15—H15B	109.5
C3—C2—H2	118.7	H15A—C15—H15B	109.5
C4—C3—C2	120.3 (2)	C1—C15—H15C	109.5
C4—C3—H3	119.9	H15A—C15—H15C	109.5
C2—C3—H3	119.9	H15B—C15—H15C	109.5
C3—C4—C5	119.0 (2)	N1—C16—C18	106.10 (17)
C3—C4—H4	120.5	N1—C16—C19	109.17 (18)
C5—C4—H4	120.5	C18—C16—C19	110.05 (19)
N3—C5—N2	111.49 (16)	N1—C16—C17	112.57 (18)
N3—C5—C4	129.27 (19)	C18—C16—C17	109.6 (2)
N2—C5—C4	119.23 (19)	C19—C16—C17	109.3 (2)
C7—C6—N1	134.20 (17)	C16—C17—H17A	109.5
C7—C6—N2	104.80 (15)	C16—C17—H17B	109.5
N1—C6—N2	120.27 (17)	H17A—C17—H17B	109.5
N3—C7—C6	111.55 (17)	C16—C17—H17C	109.5
N3—C7—C8	120.23 (17)	H17A—C17—H17C	109.5
C6—C7—C8	128.23 (17)	H17B—C17—H17C	109.5
C9—C8—C13	117.03 (18)	C16—C18—H18A	109.5
C9—C8—C7	120.86 (17)	C16—C18—H18B	109.5
C13—C8—C7	122.08 (18)	H18A—C18—H18B	109.5
C10—C9—C8	122.11 (19)	C16—C18—H18C	109.5
C10—C9—H9	118.9	H18A—C18—H18C	109.5
C8—C9—H9	118.9	H18B—C18—H18C	109.5
C11—C10—C9	119.9 (2)	C16—C19—H19A	109.5
C11—C10—H10	120.0	C16—C19—H19B	109.5
C9—C10—H10	120.0	H19A—C19—H19B	109.5
O1—C11—C10	125.4 (2)	C16—C19—H19C	109.5
O1—C11—C12	115.63 (19)	H19A—C19—H19C	109.5
C10—C11—C12	119.0 (2)	H19B—C19—H19C	109.5
C13—C12—C11	120.7 (2)		
			
C5—N2—C1—C2	−8.4 (3)	N1—C6—C7—N3	−166.5 (2)
C6—N2—C1—C2	176.4 (2)	N2—C6—C7—N3	3.3 (2)
C5—N2—C1—C15	166.7 (2)	N1—C6—C7—C8	14.0 (4)
C6—N2—C1—C15	−8.5 (3)	N2—C6—C7—C8	−176.20 (18)
N2—C1—C2—C3	2.2 (3)	N3—C7—C8—C9	29.4 (3)
C15—C1—C2—C3	−172.7 (2)	C6—C7—C8—C9	−151.2 (2)
C1—C2—C3—C4	3.7 (4)	N3—C7—C8—C13	−148.62 (19)
C2—C3—C4—C5	−3.5 (4)	C6—C7—C8—C13	30.8 (3)
C7—N3—C5—N2	−1.7 (2)	C13—C8—C9—C10	−0.8 (3)
C7—N3—C5—C4	176.8 (2)	C7—C8—C9—C10	−178.9 (2)
C1—N2—C5—N3	−172.63 (17)	C8—C9—C10—C11	0.3 (4)
C6—N2—C5—N3	3.7 (2)	C14—O1—C11—C10	−0.2 (4)
C1—N2—C5—C4	8.7 (3)	C14—O1—C11—C12	179.3 (2)
C6—N2—C5—C4	−174.96 (18)	C9—C10—C11—O1	179.6 (2)
C3—C4—C5—N3	179.1 (2)	C9—C10—C11—C12	0.0 (4)
C3—C4—C5—N2	−2.5 (3)	O1—C11—C12—C13	−179.5 (2)
C16—N1—C6—C7	75.4 (3)	C10—C11—C12—C13	0.1 (4)
C16—N1—C6—N2	−93.2 (2)	C11—C12—C13—C8	−0.6 (4)
C1—N2—C6—C7	171.71 (19)	C9—C8—C13—C12	0.9 (3)
C5—N2—C6—C7	−4.1 (2)	C7—C8—C13—C12	179.0 (2)
C1—N2—C6—N1	−16.7 (3)	C6—N1—C16—C18	170.52 (18)
C5—N2—C6—N1	167.46 (17)	C6—N1—C16—C19	52.0 (2)
C5—N3—C7—C6	−1.1 (2)	C6—N1—C16—C17	−69.6 (3)
C5—N3—C7—C8	178.47 (17)		

### N-tert-Butyl-2-(4-methoxyphenyl)-5-methylimidazo[1,2-a]pyridin-3-amine (I) Hydrogen-bond geometry (Å, º)

Cg4 is the centroid of the imidazole ring system N2/N3/C1–C7.

**Table:**

*D*—H···*A*	*D*—H	H···*A*	*D*···*A*	*D*—H···*A*
N1—H1*A*···N3^i^	0.84 (2)	2.41 (2)	3.226 (2)	163.6 (19)
C14—H14*A*···*Cg*4^ii^	0.96	2.93	3.862 (3)	165

Symmetry codes: (i) *x*, −*y*+3/2, *z*+1/2; (ii) *x*−1, *y*, *z*.

### N-tert-Butyl-2-[4-(dimethylamino)phenyl]imidazo[1,2-a]pyridin-3-amine (II) Crystal data

**Table:**

C_19_H_24_N_4_	*F*(000) = 1328
*M**_r_* = 308.42	*D*_x_ = 1.171 Mg m^−^^3^
Monoclinic, *C*2/*c*	Mo *K*α radiation, λ = 0.71073 Å
*a* = 34.9185 (14) Å	Cell parameters from 4941 reflections
*b* = 8.4656 (5) Å	θ = 2.3–21.5°
*c* = 11.8361 (6) Å	µ = 0.07 mm^−^^1^
β = 91.061 (5)°	*T* = 296 K
*V* = 3498.2 (3) Å^3^	Block, brown
*Z* = 8	0.15 × 0.10 × 0.10 mm

### N-tert-Butyl-2-[4-(dimethylamino)phenyl]imidazo[1,2-a]pyridin-3-amine (II) Data collection

**Table:**

Bruker Kappa APEXII CCD diffractometer	3259 independent reflections
Radiation source: fine-focus sealed tube	1834 reflections with *I* > 2σ(*I*)
Graphite monochromator	*R*_int_ = 0.071
ω and φ scan	θ_max_ = 25.5°, θ_min_ = 2.3°
Absorption correction: multi-scan (SADABS; Bruker, 2016)	*h* = −42→42
*T*_min_ = 0.697, *T*_max_ = 0.745	*k* = −10→10
32313 measured reflections	*l* = −14→14

### N-tert-Butyl-2-[4-(dimethylamino)phenyl]imidazo[1,2-a]pyridin-3-amine (II) Refinement

**Table:**

Refinement on *F*^2^	Secondary atom site location: difference Fourier map
Least-squares matrix: full	Hydrogen site location: mixed
*R*[*F*^2^ > 2σ(*F*^2^)] = 0.049	H atoms treated by a mixture of independent and constrained refinement
*wR*(*F*^2^) = 0.159	*w* = 1/[σ^2^(*F*_o_^2^) + (0.0675*P*)^2^ + 2.4598*P*] where *P* = (*F*_o_^2^ + 2*F*_c_^2^)/3
*S* = 1.01	(Δ/σ)_max_ = 0.001
3259 reflections	Δρ_max_ = 0.22 e Å^−^^3^
218 parameters	Δρ_min_ = −0.18 e Å^−^^3^
0 restraints	Extinction correction: SHELXL2018 (Sheldrick, 2015b), Fc^*^=kFc[1+0.001xFc^2^λ^3^/sin(2θ)]^-1/4^
Primary atom site location: structure-invariant direct methods	Extinction coefficient: 0.0021 (4)

### N-tert-Butyl-2-[4-(dimethylamino)phenyl]imidazo[1,2-a]pyridin-3-amine (II) Special details

**Table:**

Geometry. All esds (except the esd in the dihedral angle between two l.s. planes) are estimated using the full covariance matrix. The cell esds are taken into account individually in the estimation of esds in distances, angles and torsion angles; correlations between esds in cell parameters are only used when they are defined by crystal symmetry. An approximate (isotropic) treatment of cell esds is used for estimating esds involving l.s. planes.

### N-tert-Butyl-2-[4-(dimethylamino)phenyl]imidazo[1,2-a]pyridin-3-amine (II) Fractional atomic coordinates and isotropic or equivalent isotropic displacement parameters (Å^2^)

**Table:**

	*x*	*y*	*z*	*U*_iso_*/*U*_eq_	
N1	0.58246 (6)	0.2712 (2)	0.25418 (17)	0.0442 (6)	
H1A	0.5872 (7)	0.328 (3)	0.196 (2)	0.059 (8)*	
N2	0.55040 (5)	0.3250 (2)	0.42684 (15)	0.0409 (5)	
N3	0.58530 (6)	0.5154 (2)	0.50989 (15)	0.0438 (5)	
N4	0.72538 (7)	0.8380 (3)	0.2431 (2)	0.0757 (8)	
C1	0.52002 (7)	0.2237 (3)	0.4153 (2)	0.0508 (7)	
H1	0.517019	0.162595	0.350459	0.061*	
C2	0.49463 (8)	0.2134 (3)	0.4987 (2)	0.0571 (7)	
H2	0.473839	0.145323	0.491098	0.068*	
C3	0.49907 (8)	0.3043 (3)	0.5975 (2)	0.0582 (7)	
H3	0.481526	0.294650	0.655271	0.070*	
C4	0.52893 (7)	0.4063 (3)	0.6088 (2)	0.0534 (7)	
H4	0.531869	0.466408	0.674089	0.064*	
C5	0.55533 (7)	0.4205 (3)	0.52145 (18)	0.0421 (6)	
C6	0.57963 (7)	0.3602 (3)	0.35289 (18)	0.0393 (6)	
C7	0.60021 (6)	0.4794 (3)	0.40559 (18)	0.0401 (6)	
C8	0.63292 (7)	0.5687 (3)	0.36299 (18)	0.0402 (6)	
C9	0.66073 (7)	0.6297 (3)	0.4354 (2)	0.0497 (7)	
H9	0.658973	0.610551	0.512451	0.060*	
C10	0.69081 (8)	0.7177 (3)	0.3970 (2)	0.0553 (7)	
H10	0.708854	0.756665	0.448615	0.066*	
C11	0.69492 (7)	0.7499 (3)	0.2825 (2)	0.0508 (7)	
C12	0.66718 (7)	0.6877 (3)	0.2090 (2)	0.0508 (7)	
H12	0.669089	0.705457	0.131802	0.061*	
C13	0.63701 (7)	0.6006 (3)	0.24861 (19)	0.0462 (6)	
H13	0.618807	0.561903	0.197371	0.055*	
C14	0.75347 (10)	0.8995 (5)	0.3193 (3)	0.1142 (15)	
H14A	0.741602	0.971727	0.370444	0.171*	
H14B	0.772753	0.954037	0.277596	0.171*	
H14C	0.765061	0.814507	0.361250	0.171*	
C15	0.72835 (11)	0.8749 (5)	0.1261 (3)	0.1039 (13)	
H15A	0.727624	0.779098	0.082673	0.156*	
H15B	0.752067	0.928862	0.113508	0.156*	
H15C	0.707347	0.941516	0.103264	0.156*	
C16	0.60865 (8)	0.1322 (3)	0.25466 (19)	0.0507 (7)	
C17	0.64993 (10)	0.1807 (4)	0.2766 (4)	0.1032 (13)	
H17A	0.657379	0.256783	0.220981	0.155*	
H17B	0.666177	0.089525	0.272039	0.155*	
H17C	0.652367	0.226531	0.350538	0.155*	
C18	0.60542 (11)	0.0597 (4)	0.1387 (2)	0.0899 (12)	
H18A	0.579787	0.021809	0.125922	0.135*	
H18B	0.623086	−0.026745	0.133299	0.135*	
H18C	0.611317	0.137832	0.082842	0.135*	
C19	0.59690 (11)	0.0154 (4)	0.3429 (3)	0.0996 (13)	
H19A	0.597112	0.065823	0.415539	0.149*	
H19B	0.614509	−0.071669	0.344110	0.149*	
H19C	0.571574	−0.022513	0.325394	0.149*	

### N-tert-Butyl-2-[4-(dimethylamino)phenyl]imidazo[1,2-a]pyridin-3-amine (II) Atomic displacement parameters (Å^2^)

**Table:**

	*U*^11^	*U*^22^	*U*^33^	*U*^12^	*U*^13^	*U*^23^
N1	0.0602 (14)	0.0398 (12)	0.0327 (12)	0.0071 (10)	0.0018 (10)	−0.0009 (9)
N2	0.0434 (12)	0.0376 (11)	0.0417 (11)	−0.0020 (10)	0.0017 (9)	−0.0031 (9)
N3	0.0492 (12)	0.0451 (12)	0.0372 (11)	−0.0053 (10)	0.0044 (9)	−0.0036 (9)
N4	0.0694 (17)	0.0800 (19)	0.0784 (18)	−0.0236 (15)	0.0188 (14)	0.0092 (14)
C1	0.0549 (16)	0.0452 (15)	0.0524 (16)	−0.0078 (13)	−0.0015 (13)	−0.0073 (12)
C2	0.0529 (17)	0.0563 (17)	0.0623 (17)	−0.0120 (14)	0.0069 (14)	−0.0029 (14)
C3	0.0564 (17)	0.0631 (18)	0.0556 (17)	−0.0070 (15)	0.0144 (13)	0.0000 (14)
C4	0.0596 (17)	0.0574 (17)	0.0434 (14)	−0.0049 (15)	0.0107 (12)	−0.0057 (13)
C5	0.0490 (15)	0.0422 (14)	0.0350 (13)	−0.0032 (12)	0.0017 (11)	−0.0043 (11)
C6	0.0462 (14)	0.0375 (13)	0.0343 (12)	0.0014 (11)	0.0017 (11)	−0.0019 (10)
C7	0.0438 (14)	0.0400 (13)	0.0365 (13)	0.0026 (11)	0.0020 (10)	0.0013 (11)
C8	0.0437 (14)	0.0388 (13)	0.0384 (13)	0.0017 (12)	0.0037 (10)	−0.0016 (11)
C9	0.0542 (16)	0.0538 (16)	0.0411 (14)	−0.0058 (14)	0.0017 (12)	−0.0010 (12)
C10	0.0514 (16)	0.0569 (17)	0.0575 (17)	−0.0098 (14)	0.0015 (13)	−0.0036 (13)
C11	0.0501 (16)	0.0431 (15)	0.0596 (17)	−0.0023 (13)	0.0114 (13)	−0.0004 (13)
C12	0.0621 (17)	0.0482 (15)	0.0426 (14)	0.0011 (14)	0.0128 (13)	0.0041 (12)
C13	0.0516 (16)	0.0464 (15)	0.0407 (14)	−0.0017 (13)	0.0015 (11)	0.0005 (11)
C14	0.082 (3)	0.131 (4)	0.129 (3)	−0.059 (3)	−0.003 (2)	0.019 (3)
C15	0.107 (3)	0.115 (3)	0.092 (3)	−0.034 (2)	0.043 (2)	0.006 (2)
C16	0.0687 (18)	0.0412 (14)	0.0422 (14)	0.0131 (13)	0.0054 (12)	−0.0005 (11)
C17	0.074 (2)	0.082 (3)	0.154 (4)	0.029 (2)	−0.012 (2)	−0.021 (2)
C18	0.145 (3)	0.071 (2)	0.0538 (18)	0.040 (2)	0.0069 (19)	−0.0116 (16)
C19	0.150 (3)	0.066 (2)	0.084 (2)	0.046 (2)	0.043 (2)	0.0315 (19)

### N-tert-Butyl-2-[4-(dimethylamino)phenyl]imidazo[1,2-a]pyridin-3-amine (II) Geometric parameters (Å, º)

**Table:**

N1—C6	1.395 (3)	C10—C11	1.393 (3)
N1—C16	1.491 (3)	C10—H10	0.9300
N1—H1A	0.86 (3)	C11—C12	1.393 (4)
N2—C1	1.369 (3)	C12—C13	1.375 (3)
N2—C6	1.389 (3)	C12—H12	0.9300
N2—C5	1.389 (3)	C13—H13	0.9300
N3—C5	1.329 (3)	C14—H14A	0.9600
N3—C7	1.383 (3)	C14—H14B	0.9600
N4—C11	1.387 (3)	C14—H14C	0.9600
N4—C14	1.419 (4)	C15—H15A	0.9600
N4—C15	1.425 (4)	C15—H15B	0.9600
C1—C2	1.342 (4)	C15—H15C	0.9600
C1—H1	0.9300	C16—C19	1.500 (4)
C2—C3	1.406 (4)	C16—C18	1.506 (4)
C2—H2	0.9300	C16—C17	1.516 (4)
C3—C4	1.358 (4)	C17—H17A	0.9600
C3—H3	0.9300	C17—H17B	0.9600
C4—C5	1.403 (3)	C17—H17C	0.9600
C4—H4	0.9300	C18—H18A	0.9600
C6—C7	1.381 (3)	C18—H18B	0.9600
C7—C8	1.467 (3)	C18—H18C	0.9600
C8—C9	1.383 (3)	C19—H19A	0.9600
C8—C13	1.390 (3)	C19—H19B	0.9600
C9—C10	1.372 (3)	C19—H19C	0.9600
C9—H9	0.9300		
			
C6—N1—C16	118.45 (19)	C13—C12—C11	121.2 (2)
C6—N1—H1A	112.8 (17)	C13—C12—H12	119.4
C16—N1—H1A	108.6 (17)	C11—C12—H12	119.4
C1—N2—C6	130.4 (2)	C12—C13—C8	121.9 (2)
C1—N2—C5	121.9 (2)	C12—C13—H13	119.0
C6—N2—C5	107.55 (18)	C8—C13—H13	119.0
C5—N3—C7	105.59 (18)	N4—C14—H14A	109.5
C11—N4—C14	120.6 (3)	N4—C14—H14B	109.5
C11—N4—C15	121.0 (3)	H14A—C14—H14B	109.5
C14—N4—C15	118.4 (3)	N4—C14—H14C	109.5
C2—C1—N2	119.2 (2)	H14A—C14—H14C	109.5
C2—C1—H1	120.4	H14B—C14—H14C	109.5
N2—C1—H1	120.4	N4—C15—H15A	109.5
C1—C2—C3	120.8 (3)	N4—C15—H15B	109.5
C1—C2—H2	119.6	H15A—C15—H15B	109.5
C3—C2—H2	119.6	N4—C15—H15C	109.5
C4—C3—C2	120.1 (2)	H15A—C15—H15C	109.5
C4—C3—H3	119.9	H15B—C15—H15C	109.5
C2—C3—H3	119.9	N1—C16—C19	110.3 (2)
C3—C4—C5	119.7 (2)	N1—C16—C18	106.4 (2)
C3—C4—H4	120.1	C19—C16—C18	110.4 (3)
C5—C4—H4	120.1	N1—C16—C17	111.6 (2)
N3—C5—N2	110.80 (19)	C19—C16—C17	109.3 (3)
N3—C5—C4	131.1 (2)	C18—C16—C17	108.7 (3)
N2—C5—C4	118.1 (2)	C16—C17—H17A	109.5
C7—C6—N2	104.74 (19)	C16—C17—H17B	109.5
C7—C6—N1	136.7 (2)	H17A—C17—H17B	109.5
N2—C6—N1	118.4 (2)	C16—C17—H17C	109.5
C6—C7—N3	111.3 (2)	H17A—C17—H17C	109.5
C6—C7—C8	128.5 (2)	H17B—C17—H17C	109.5
N3—C7—C8	120.1 (2)	C16—C18—H18A	109.5
C9—C8—C13	116.5 (2)	C16—C18—H18B	109.5
C9—C8—C7	121.5 (2)	H18A—C18—H18B	109.5
C13—C8—C7	122.0 (2)	C16—C18—H18C	109.5
C10—C9—C8	122.1 (2)	H18A—C18—H18C	109.5
C10—C9—H9	119.0	H18B—C18—H18C	109.5
C8—C9—H9	119.0	C16—C19—H19A	109.5
C9—C10—C11	121.5 (2)	C16—C19—H19B	109.5
C9—C10—H10	119.3	H19A—C19—H19B	109.5
C11—C10—H10	119.3	C16—C19—H19C	109.5
N4—C11—C10	121.7 (2)	H19A—C19—H19C	109.5
N4—C11—C12	121.5 (2)	H19B—C19—H19C	109.5
C10—C11—C12	116.8 (2)		
			
C6—N2—C1—C2	−178.1 (2)	C5—N3—C7—C6	−0.7 (3)
C5—N2—C1—C2	−1.4 (4)	C5—N3—C7—C8	177.1 (2)
N2—C1—C2—C3	−0.5 (4)	C6—C7—C8—C9	−150.6 (2)
C1—C2—C3—C4	1.2 (4)	N3—C7—C8—C9	32.0 (3)
C2—C3—C4—C5	0.0 (4)	C6—C7—C8—C13	31.5 (4)
C7—N3—C5—N2	−0.4 (2)	N3—C7—C8—C13	−145.9 (2)
C7—N3—C5—C4	−178.6 (3)	C13—C8—C9—C10	0.2 (4)
C1—N2—C5—N3	−175.9 (2)	C7—C8—C9—C10	−177.9 (2)
C6—N2—C5—N3	1.4 (3)	C8—C9—C10—C11	−0.1 (4)
C1—N2—C5—C4	2.5 (3)	C14—N4—C11—C10	−0.4 (4)
C6—N2—C5—C4	179.9 (2)	C15—N4—C11—C10	−177.6 (3)
C3—C4—C5—N3	176.3 (3)	C14—N4—C11—C12	−179.6 (3)
C3—C4—C5—N2	−1.8 (4)	C15—N4—C11—C12	3.2 (4)
C1—N2—C6—C7	175.3 (2)	C9—C10—C11—N4	−179.6 (3)
C5—N2—C6—C7	−1.7 (2)	C9—C10—C11—C12	−0.4 (4)
C1—N2—C6—N1	−7.4 (4)	N4—C11—C12—C13	−179.9 (2)
C5—N2—C6—N1	175.56 (19)	C10—C11—C12—C13	0.8 (4)
C16—N1—C6—C7	81.8 (4)	C11—C12—C13—C8	−0.8 (4)
C16—N1—C6—N2	−94.4 (3)	C9—C8—C13—C12	0.3 (4)
N2—C6—C7—N3	1.6 (3)	C7—C8—C13—C12	178.3 (2)
N1—C6—C7—N3	−175.0 (2)	C6—N1—C16—C19	59.6 (3)
N2—C6—C7—C8	−176.1 (2)	C6—N1—C16—C18	179.4 (2)
N1—C6—C7—C8	7.4 (4)	C6—N1—C16—C17	−62.1 (3)

### N-tert-Butyl-2-[4-(dimethylamino)phenyl]imidazo[1,2-a]pyridin-3-amine (II) Hydrogen-bond geometry (Å, º)

Cg3 is the centroid of benzene ring C8–C13.

**Table:**

*D*—H···*A*	*D*—H	H···*A*	*D*···*A*	*D*—H···*A*
N1—H1*A*···N3^i^	0.86 (3)	2.56 (3)	3.412 (3)	167 (2)
C13—H13···N3^i^	0.93	2.57	3.467 (3)	161
C19—H19*B*···*Cg*3^ii^	0.96	2.87	3.829 (4)	174

Symmetry codes: (i) *x*, −*y*+1, *z*−1/2; (ii) *x*, *y*−1, *z*.
